# Laparoscopic Excision of a Psoas Epidermoid Cyst: A Report of a Rare Case

**DOI:** 10.7759/cureus.98810

**Published:** 2025-12-09

**Authors:** Fiza M Dar, Simon Hakobyan, Amirah I Bhatti, Manzoor A Dar

**Affiliations:** 1 Cardiology, University Hospitals Coventry and Warwickshire, Coventry, GBR; 2 Accident and Emergency, St John's Hospital, Livingston, GBR; 3 Palliative Care, Royal Berkshire Hospital, Reading, GBR; 4 General Surgery, Saada Hospital, Sohar, OMN

**Keywords:** epidermoid cyst, epidermoid psoas cyst, gharbi, gonzalez-crussi, hydatid cyst, laparoscopy, retroperitoneal cyst

## Abstract

Retroperitoneal cysts are rare, and retroperitoneal epidermoid cysts are fewer in number; these cysts often pose challenges in being diagnostically vague. This report presents a case of a 41-year-old man who presented with dysuria and bilateral loin pain, and was treated for bilateral ureteric calculi shown on an abdominal X-ray. The patient re-presented with pain, and further imaging revealed a provisional diagnosis of a hydatid cyst, demonstrated by a large mass within the right psoas muscle. Histopathology after a complete laparoscopic excision confirmed a benign epidermoid cyst (Gonzalez-Crussi grade 0). This case demonstrates that retroperitoneal cysts pose a diagnostic challenge due to vague symptoms and multiple plausible differential diagnoses. A multimodal evaluation and complete surgical excision have proven to be therapeutically vital, with a minimal rate of recurrence.

## Introduction

Retroperitoneal cysts are rare, with retroperitoneal epidermoid cysts accounting for less than 1% among these, and cysts in the psoas compartment more infrequent [1]. Histologically, epidermoid cysts are keratin-filled and lined by stratified squamous epithelium [2] with no skin that distinguishes them from dermoid cysts. The origin of these cysts is still unclear; however, there are theories of abnormal sequestration in the retroperitoneal space during embryological development or of them being acquired or traumatic. However, this is more likely for other body parts, such as cysts on the skin [3]. Patients are typically asymptomatic, and the cyst is an incidental finding, or patients may present with vague symptoms such as abdominal pain and distension from a mass effect [4].

Differential diagnoses include hydatid cysts (mimic epidermoid cysts as well-circumscribed unilocular cysts), abscesses (or a psoas abscess in this case due to thick enhancing walls and surrounding inflammation), germ cell tumors (as both may contain keratinaceous debris and squamous epithelium) [4], and mucinous cystic neoplasms (also unilocular cysts); these overlaps and similarities warrant a multimodal approach with appropriate imaging, serology, surgery, and histopathology. Surgery and histopathological examination of the lesion remain the gold standard for diagnosis and treatment. Due to the lack of literature, the vague nature of symptoms, and the range of mimicked conditions, a diagnostic and curative challenge is faced [5]. This case report presents a rare case of a complete laparoscopic excision of a psoas epidermoid cyst and is one of the only cases reported in English literature. It highlights the importance of a multimodal approach, careful and complete surgical excision, and histological diagnosis in successful treatment.

## Case presentation

A 41-year-old man with a past medical history of hypertension and polyuria presented to the emergency department with bilateral flank pain and dysuria. An abdominal X-ray showed bilateral ureteric calculi, and hence, the patient was referred to urology. An ultrasound four days later revealed a large mass measuring 13 x 20 cm adjacent to the left lobe of the liver and pancreas with no vascularity (there is no stored image of this). The patient was referred to general surgery but, in the interim of being seen, re-presented to the emergency department 21 days later with bilateral flank pain and new-onset epigastric discomfort. Examination revealed abdominal fullness with no palpable mass and mild epigastric tenderness.

As seen in Table 1, initial investigations, including a full blood count, liver and renal function tests, alpha-fetoprotein (AFP), cancer antigen 19-9 (CA 19-9), erythrocyte sedimentation rate (ESR), antibody screening, purified protein derivative, and *Echinococcus* serology (to rule out a hydatid cyst), were unremarkable.

**Table 1:** Pre-operative laboratory findings ALT: alanine transaminase, ALP: alkaline phosphatase, AFP: alpha-fetoprotein, CA 19-9: cancer antigen 19-9, ESR: erythrocyte sedimentation rate, PPD: purified protein derivative, IgG: immunoglobulin G

Parameter	Result	Reference range	Units
Hemoglobin	132	130-170	g/L
White cell count	8.44	4-11	×10^9^/L
C-reactive protein	10	<4	mg/L
Urea	7.4	2.5-7.8	mmol/L
Creatinine	96	59-104	umol/L
ALT	45	10-49	U/L
ALP	78	30-130	U/L
AFP	Negative, 8	0-10	ng/mL
CA 19-9	Negative, 30	0-37	U/mL
ESR	3	0-15 (for age)	mm/hour
*Echinococcus* IgG	Negative	-	-
PPD	Negative	-	-

**Figure 1:**
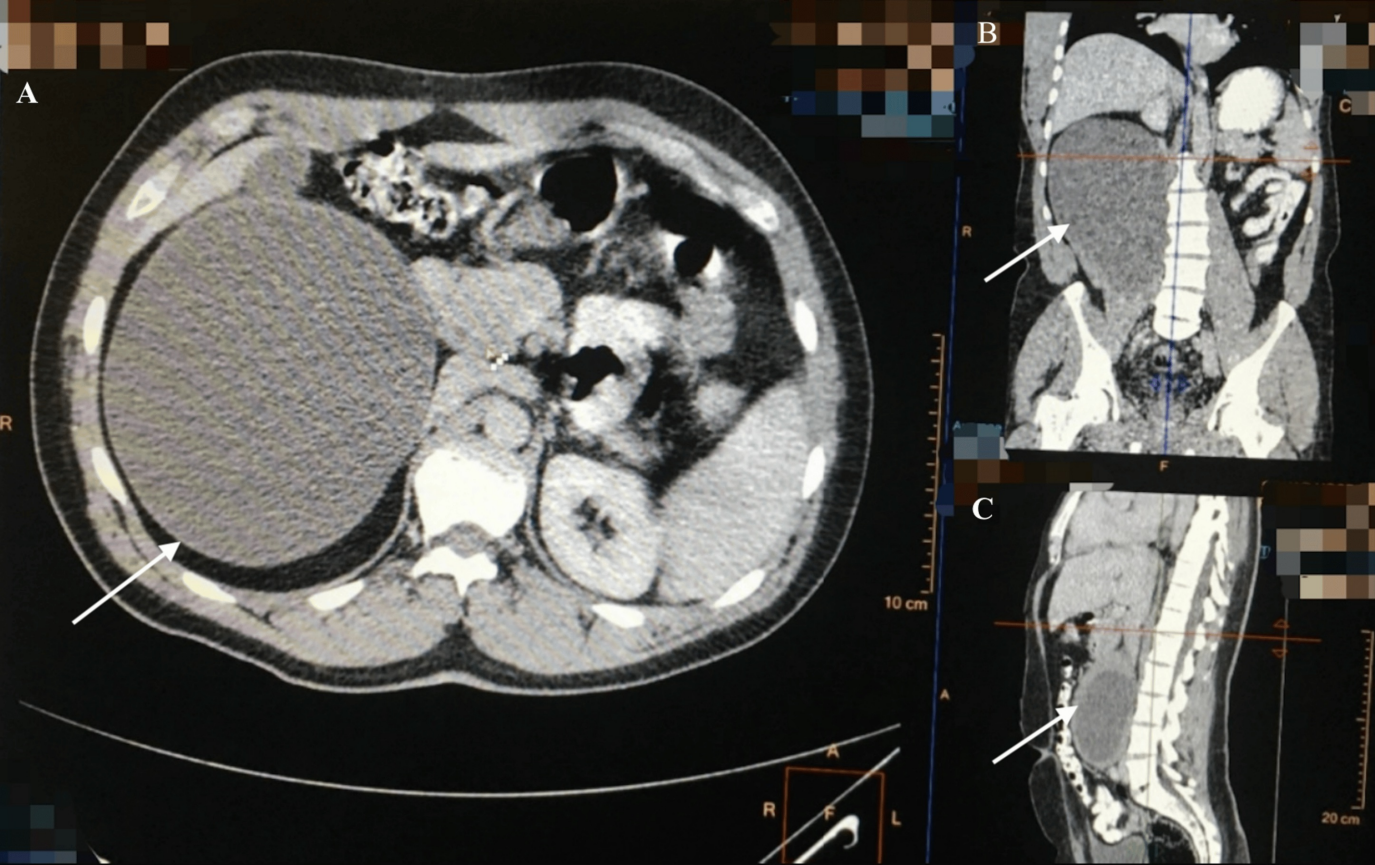
CT image demonstrating a large, well-defined cystic lesion (20 x 14 cm) within the right psoas muscle (arrows) (A) Axial view. (B) Coronal view. (C) Sagittal view. CT: computed tomography

**Figure 2:**
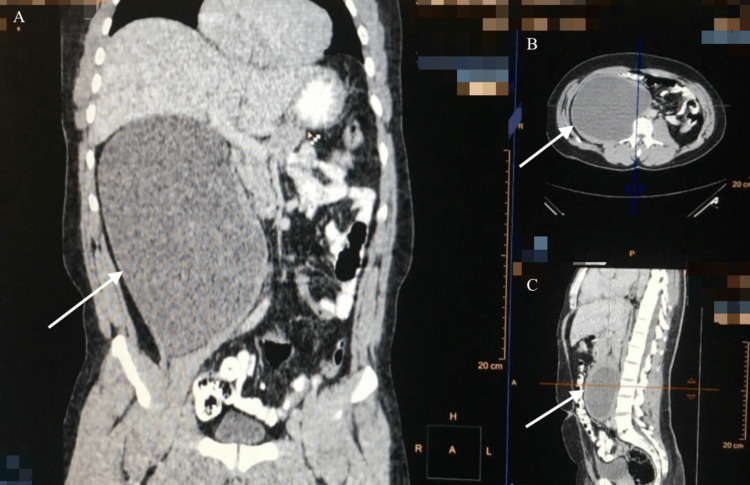
CT image demonstrating a large, well-defined cystic lesion (20 x 14 cm) within the right psoas muscle (arrows) (A) Coronal view. (B) Axial view. (C) Sagittal view. CT: computed tomography

**Figure 3:**
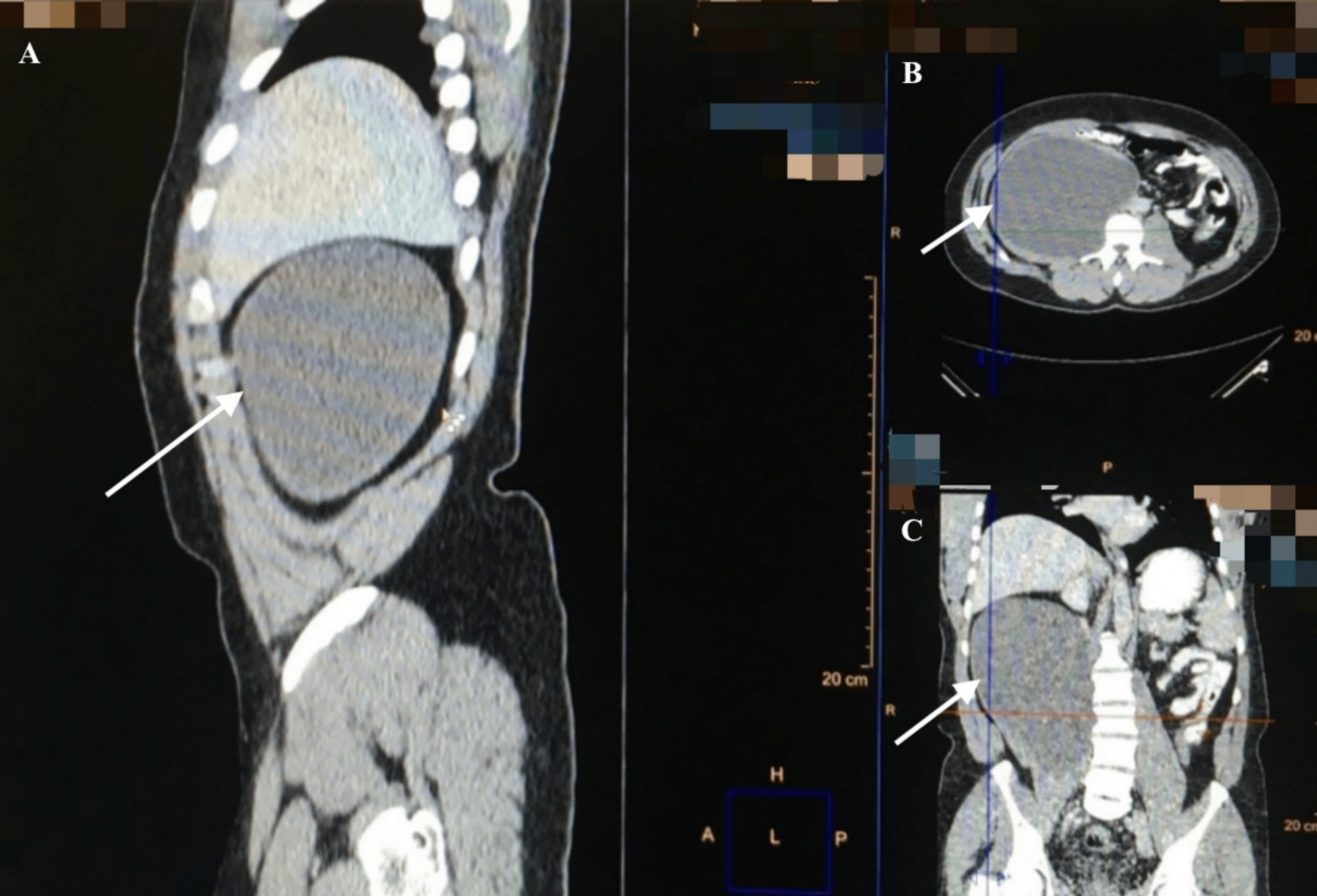
CT image demonstrating a large, well-defined cystic lesion (20 x 14 cm) within the right psoas muscle (arrows) (A) Sagittal view. (B) Axial view. (C) Coronal view. CT: computed tomography

**Figure 4:**
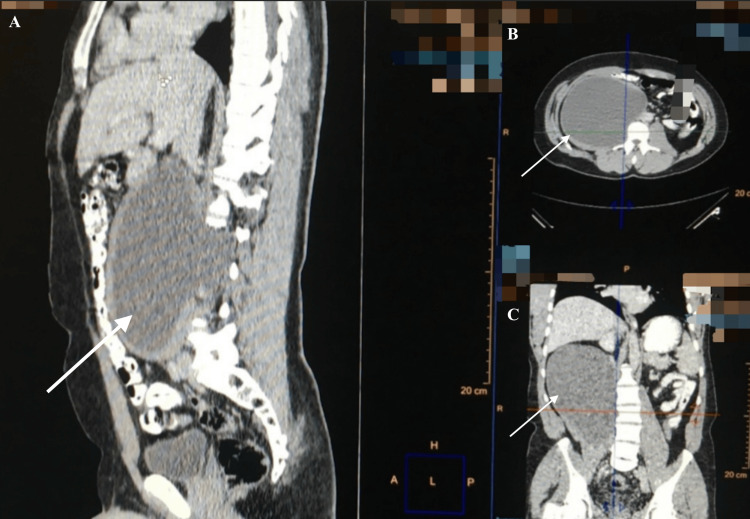
CT image demonstrating a large, well-defined cystic lesion (20 x 14 cm) within the right psoas muscle (arrows) (A) Sagittal view. (B) Axial view. (C) Coronal view. CT: computed tomography

An abdominal and pelvic computed tomography (CT) two days later demonstrated a cystic mass measuring 14 x 20 cm within the right psoas muscle, suggestive of a hydatid cyst (Figures 1-4).

**Figure 5:**
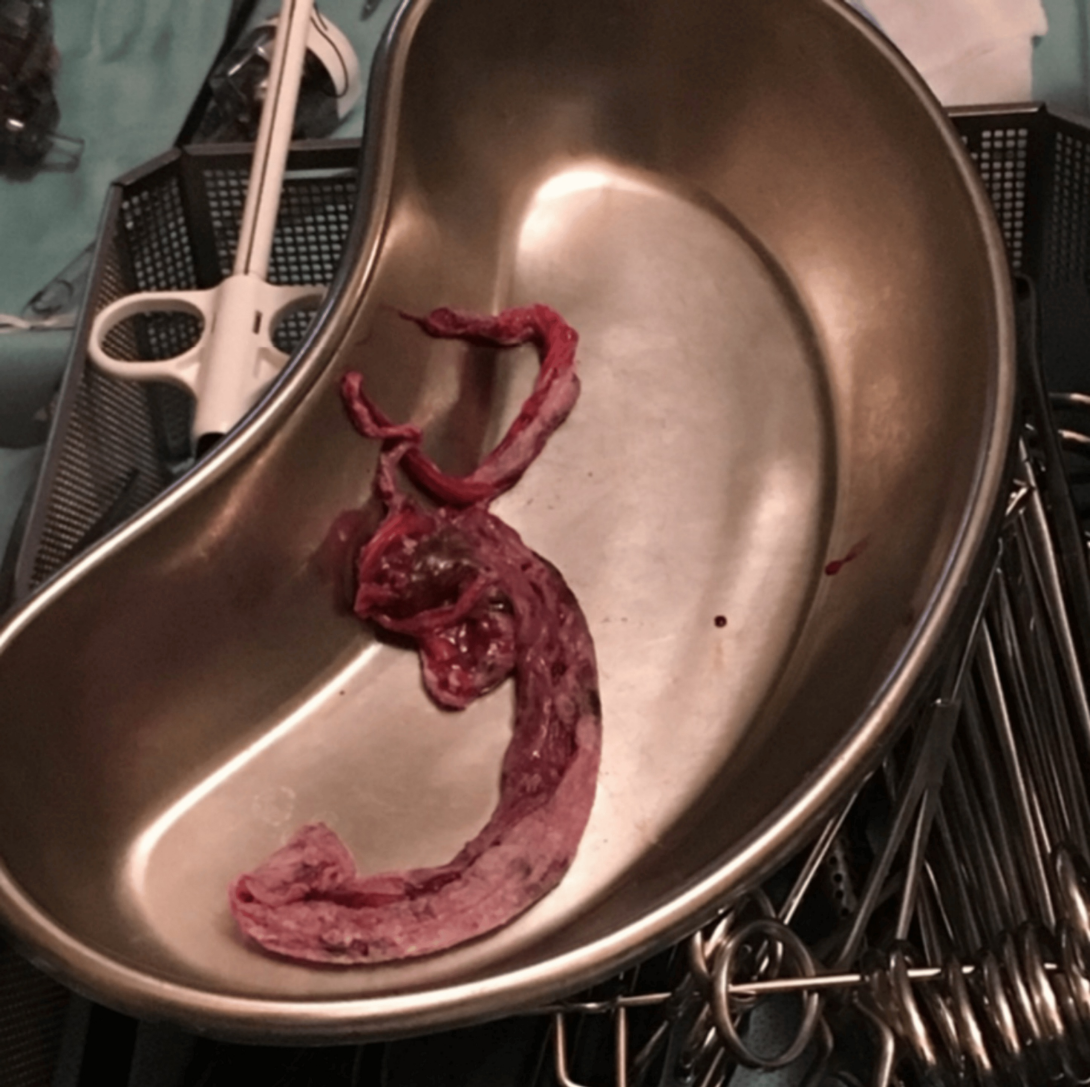
Cyst capsule showing excision en toto

The patient underwent a retroperitoneal laparoscopic exploration, and the cyst was excised en toto. The procedure was done under general anesthesia in the left lateral position. The first incision was made 4 cm anterior and 2 cm above the anterior superior iliac spine, and this was deepened with sharp and blunt dissection to create a pre-peritoneal space. A 10 mm trocar was introduced (Video 1), and further dissection of the pre-peritoneal space was carried out using the telescope (Video 2). Another 10 mm trocar was introduced as the posterior axillary line, midway between the costal margin and iliac crest. A third 5 mm trocar was introduced at a forefinger breadth above the anterior abdominal incision. The space along the psoas muscle was widened, and the 10 mm was directly introduced into the cyst due to the large size and to minimize manipulation. From this, thick white material (resembling keratinous material) was aspirated (Video 3), and the cavity was irrigated with saline until approximately 60% of the contents were evacuated. The cyst wall was dissected with blunt and sharp dissection (Video 4) using a diathermy and, while particularly adherent at the superior and inferior poles, was excised en toto (Figure 5). The space was irrigated with saline, and a Redivac drain was inserted. All incisions were closed in layers with vicryl, and the skin was closed with 3-0 nylon.

**Video 1:** Incision made 4 cm anterior and 2 cm above the anterior superior iliac spine and deepened with sharp and blunt dissection, followed by the insertion of a 10 mm trocar The patient was positioned in the left lateral position.

**Video 2:** Further dissection of the pre-peritoneal space via telescope This video depicts the pre-peritoneal space with the psoas muscle positioned inferiorly.

**Video 3:** Intra-operative video depicting the trocar inserted into the cyst through the psoas muscle and suction of thick, white keratinous fluid from the cyst cavity The psoas muscle is positioned inferiorly.

**Video 4:** Video depicting the cyst wall after cyst contents were evacuated and dissection of the cyst out of the psoas muscle using blunt and sharp dissection The letters “KARL STORZ” represent the company Karl Storz, a medical instrument manufacturer. The psoas muscle is inferior, and a gauze is seen superior.

Post-operatively, the patient experienced shoulder-tip pain, attributed to diaphragmatic irritation, and constipation, which were managed conservatively. The patient received albendazole for seven days, followed by cefuroxime and metronidazole for a duration of seven days for surgical prophylaxis and low-molecular-weight heparin for five days for thromboprophylaxis. Post-operative investigations showed transient leukocytosis (Table 2), and other blood tests remained stable. The drain was removed three days post-operatively, and an ultrasound six days post-operatively confirmed no intra-abdominal free fluid, and the patient was discharged. The patient was followed up at six months and remained asymptomatic, after which the patient was lost to follow-up.

**Table 2:** Post-operative laboratory findings ALT: alanine transaminase, ALP: alkaline phosphatase

Parameter	Result	Reference range	Units
Hemoglobin	128	130-170	g/L
White cell count	12.38	4-11	×10^9^/L
C-reactive protein	21	<4	mg/L
Urea	7.8	2.5-7.8	mmol/L
Creatinine	99	59-104	umol/L
ALT	44	10-49	U/L
ALP	75	30-130	U/L

A microbiological culture and sensitivity of the cyst fluid showed no bacterial growth. Cytology revealed plenty of anucleate and mature nucleated squamous cells. Histopathology confirmed a benign epidermoid cyst classified as Gharbi-1 (unilocular cyst) because hydatid disease was suspected, although this system does not apply to epidermoid cysts. It was also staged as TNRC: T - psoas, N 0-1, R 0, C1 (tumor in psoas, no/possible nodal involvement, complete resection, cyst type 1). No immature elements were identified, corresponding to Gonzalez-Crussi grade 0 (mature, benign).

At one year post-operative, the patient remained asymptomatic with normal tumor markers and imaging.

## Discussion

Retroperitoneal cysts are rare, with retroperitoneal epidermoid cysts comprising less than 1% of retroperitoneal masses [1]. At the time of writing this article, there is limited known published work on a psoas epidermoid cyst, and given its rarity, this case report contributes to the limited literature on the treatment of this subtype.

There are two main theories pertaining to sequestration in the retroperitoneal space during embryological development or cysts being a result of trauma/acquisition, with the latter more likely in locations such as the skin [3]. Based on embryological and histological differentiation, retroperitoneal cysts can be classified into the categories of “(a) urogenital, (b) mesocolic, (c) cysts arising in cell inclusions, (d) traumatic, (e) parasitic, and (f) lymphatic” [6]. In this case, the cyst did not classify in the above categories. Retroperitoneal cysts can also be classified as neoplastic and nonneoplastic, and in this case, the cyst was benign. Around one-third of cases are found incidentally, with patients remaining largely asymptomatic. Patients often present with vague symptoms such as loin pain or a mass effect [4], and in this case, symptoms such as loin pain and dysuria led to a delayed appropriate recognition of an underlying mass and initial treatment for ureteric calculi.

Imaging modalities such as ultrasound, computed tomography (CT), and magnetic resonance imaging (MRI) are vital in characterizing the cyst [7]. Ultrasounds are useful as initial low-risk imaging, although they are limited in terms of characterization and by retroperitoneal structures and bowel gas [8]. On CT, epidermoid cysts appear as unilocular, well-defined, and thin-walled cysts [4], although this can overlap with hydatid cysts, which also have a unilocular appearance. MRI is useful in defining soft tissue planes and visualizing any local invasion [7].

In this case, an overlap with a hydatid cyst is seen; however, negative *Echinococcus* serology (sensitive in 70%-90% of hydatid cysts) [9] and other findings show the importance of histopathology remaining the gold standard for diagnosis.

In histopathology, epidermoid cysts are seen lined by stratified squamous epithelium with lamellated keratin in the lumen [2]; unfortunately, specimen imaging from this case is not available.

Key differential diagnoses for a cystic mass in the psoas region include, but are not limited to, (1) non-neoplastic lesions including a psoas abscess, hydatid cyst, hematoma, lipoma, and abdominal aortic aneurysm, and (2) neoplastic lesions including germ cell tumors (e.g., teratoma), paraganglioma, leiomyosarcoma, pancreatic pseudocyst, and a mucinous cystadenoma [4].

Epidermoid cysts must be differentiated from teratomas using histopathology, and it must be noted that immature teratomas are prone to malignancy [10]. At the time of this case, the Gonzalez-Crussi and Gharbi system were used to stage the cyst.

The Gonzalez-Crussi system grades mature and immature teratomas, and grades are as follows: grade 0 (no immature tissue, i.e., mature and benign), grade 1 (<10% immature tissue), grade 2 (10%-50% immature tissue), and grade 3 (>50% immature tissue) [11]. In this case, the Gonzalez-Crussi grade was 0, confirming that this is benign.

The Gharbi classification system is an ultrasound-based system used for classifying hepatic hydatid cysts and splits them into five types depending on the cyst wall, contents, sonographic appearance, and viability [12]. In this case, the cyst was type 1, intact and unilocular, and hence, as per the staging system, was active and treated with albendazole [13].

According to updated English literature, there is no specialized classification used for retroperitoneal epidermoid cysts; hence, the above was used.

Tumor markers such as AFP and b-HCG are useful in predicting prognosis for germ cell tumors, with elevated levels indicating immaturity and malignancy [14]. In this case, normal tumor markers also affirmed the benign nature of the cyst.

Surgical excision and laparoscopic resection, as performed in this case, are the treatment of choice and are seen to be curative. A laparoscopic method is favored in view of its minimally invasive approach, reduced morbidity, and shorter hospital stays. In larger or technically challenging adherent lesions, a laparotomy may be required [15]. Regardless of the method, a complete excision is essential, and aspiration or marsupialization should be avoided due to the risk of recurrence and infection [16]. Complications of a surgical excision include rupture and subsequent infection and peritonitis, recurrence of retroperitoneal epidermoid cysts (1%-5%), and malignant transformation [4]. It is recommended to follow up with an ultrasound or CT to document resolution [16].

Other recent case reports, including recent case reports, echo the above in terms of surgical excision remaining definitive and highlight the importance of having a high index of suspicion, appropriate imaging, and histopathological evidence for appropriate diagnosis [17].

## Conclusions

The case presented illustrates the rarity of a psoas epidermoid cyst and the challenge associated with diagnosis (mimicking a hydatid cyst), which was solved by a complete laparoscopic excision and histopathological diagnosis confirmation. It can be noted that there is no specific grading system for retroperitoneal epidermoid cysts, given the rarity, and even more so for retroperitoneal epidermoid psoas cysts. The importance of a multimodal evaluation with imaging, serology, tumor markers, surgery, and histopathology is seen with a surgical excision often being curative.

There are a few limitations to be noted in this article, including the quality of images and the lack of stored ultrasound imaging; this is due to the retrospective nature of the report and case presented in 2016. This also affects the conclusions justified as the final diagnosis is heavily reliant on histopathology, and the lack of evidence of this creates a gap. Lastly, a further limitation is the lack of classification systems applicable to epidermoid cysts specifically, which contributed to initial diagnostic uncertainty and highlights the need for the development of an appropriate classification system.
